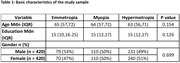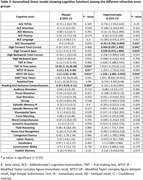# A Study on Refractive Errors and Cognitive functions in the Aging Population

**DOI:** 10.1002/alz.091233

**Published:** 2025-01-03

**Authors:** Goutham Velavarajan, Amitha C M, Ajith Partha, Divya N M, Meghana R, Meenakshi Menon, Abhishek Mensegere Lingegodwa, Albert Stezin, Monisha S, Dev Kumar HS, Rajitha Narayanasamy, Vindhya Vishwanath, Palash K Malo, Prathima Arvind, Shafeeq K Shahul Hameed, Sunitha HS, Sadhana Singh, Banashree Mondal, Deepashri Agrawal, Jonas S. Sundarakumar, Thomas Gregor Issac

**Affiliations:** ^1^ Centre for Brain Research, Indian Institute of Science, Bangalore, Karnataka India; ^2^ CBR, IISc, Bangalore, Karnataka India; ^3^ Center for Brain Research, IISc, bangalore, Karnataka India; ^4^ Centre for Brain Research, Bangalore, Karnataka India; ^5^ Centre for Brain Research, Indian Institute of Science, Bengaluru, Karnataka India

## Abstract

**Background:**

Refractive errors are common visual comorbidities among the elderly. Cognitive dysfunction also occurs in this population. A study by Ong et al (2013) demonstrated an association of refractive errors with poor cognitive performance. In this study, we explored the potential association between refractive errors (RE) and cognitive functions (CF) in an elderly population from South India.

**Method:**

This cross‐sectional analysis, based on the Tata Longitudinal Study on Aging (CBR‐TLSA) cohort (Sundarakumar et al., 2020), included participants with a minimum visual acuity of ≤ 0.52 LogMar using the LogMAR chart (Aurolab, India). Those meeting the criteria underwent objective refraction with a retinoscope (Welch Allyn, Dublin, USA) to classify RE (Emmetropia, Myopia, Hypermetropia). CF was assessed using Addenbrooke’s Cognitive Examination III (ACE III) and various cognitive tests, including Digit Forward/Backward, Trail Making Test A/B, Modified Taylor Complex Figure Immediate/Delayed Recall, Digit Simple Substitution Test (DSST), and COGNITO (Computerized assessment of adult information processing) battery domain‐specific tests. Cognitive tasks were performed post‐acuity correction with prescription glasses.

**Result:**

The demographic characteristics of the study sample are mentioned in Table 1. Among 856 participants, 149 (17%) were Emmetropes, 224 (26%) were Myopes, and 483 (56%) were Hypermetropes. The Myopes performed poorly in TMT‐B (β = 14.9 (CI: 1.16, 28.68), p = 0.03), MTCF IR (β = ‐2.15 (CI: ‐3.8, ‐0.49), p = 0.01), MTCF DR (β = ‐2.2 (CI: ‐3.9, ‐0.48), p = 0.011), and reading and sentence comprehension (β = ‐0.37 (CI: ‐0.63, ‐0.11), p = 0.004) compared to emmetropes. Hypermetropic showed poor performance in Ace visuo‐spatial (β = ‐0.43 (CI: ‐0.79, ‐0.06), p = 0.02), MTCF DR (β = ‐1.5 (CI: ‐3.08, ‐0.03), p = 0.045), and showed better performance in Digit Forward Total Correct (β = 0.96 (CI:0.03,1.89), p = 0.04) & span (β = 0.53 (CI:0.07,1.004), p = 0.02).

**Conclusion:**

Our study found that individuals with myopia and hypermetropia performed worse on certain cognitive tests compared to those with normal vision. Myopic individuals struggled with tasks involving complex figure recall and comprehension, while hypermetropic individuals had difficulties with visuospatial tasks and they did better in attention tasks. These findings suggest that corrected RE in older adults could contribute to cognitive challenges and needs to be further researched.